# Imaging Surrogates of Disease Activity in Neuromyelitis Optica Allow Distinction from Multiple Sclerosis

**DOI:** 10.1371/journal.pone.0137715

**Published:** 2015-09-18

**Authors:** Lucy Matthews, Shannon Kolind, Alix Brazier, Maria Isabel Leite, Jonathan Brooks, Anthony Traboulsee, Mark Jenkinson, Heidi Johansen-Berg, Jacqueline Palace

**Affiliations:** 1 Nuffield Department of Clinical Neurosciences, University of Oxford, Oxford, United Kingdom; 2 Neurology Department, Oxford University Hospitals NHS Trust, Oxford, United Kingdom; 3 Division of Neurology, Department of Medicine, University of British Columbia, Vancouver, Canada; 4 Clinical Research and Imaging Centre (CRiCBristol), University of Bristol, Bristol, United Kingdom; Medical University Vienna, Center for Brain Research, AUSTRIA

## Abstract

Inflammatory demyelinating lesions of the central nervous system are a common feature of both neuromyelitis optica and multiple sclerosis. Despite this similarity, it is evident clinically that the accumulation of disability in patients with neuromyelitis optica is relapse related and that a progressive phase is very uncommon. This poses the question whether there is any pathological evidence of disease activity or neurodegeneration in neuromyelitis optica between relapses. To investigate this we conducted a longitudinal advanced MRI study of the brain and spinal cord in neuromyelitis optica patients, comparing to patients with multiple sclerosis and controls. We found both cross-sectional and longitudinal evidence of diffusely distributed neurodegenerative surrogates in the multiple sclerosis group (including thalamic atrophy, cervical cord atrophy and progressive widespread diffusion and myelin water imaging abnormalities in the normal appearing white matter) but not in those with neuromyelitis optica, where localised abnormalities in the optic radiations of those with severe visual impairment were noted. In addition, between relapses, there were no new silent brain lesions in the neuromyelitis optica group. These findings indicate that global central nervous system neurodegeneration is not a feature of neuromyelitis optica. The work also questions the theory that neurodegeneration in multiple sclerosis is a chronic sequela to prior inflammatory and demyelinating pathology, as this has not been found to be the case in neuromyelitis optica where the lesions are often more destructive.

## Introduction

Neuromyelitis optica is a rare severe relapsing inflammatory central nervous system disorder that is typically focused on the spinal cord and optic nerves [1]. The associated demyelination led it to be previously classified as a variant of multiple sclerosis. More recent evidence suggests it is a primary astrocytopathy [2, 3] consequent to the discovery of pathogenic antibodies to aquaporin-4 water channels that are concentrated on the foot processes of astrocytes [4, 5]. These antibodies can be detected in the serum of the majority of neuromyelitis optica patients and appear highly specific for this disorder [6]. However not all patients have this antibody, and some patients are difficult to distinguish from multiple sclerosis.

In contrast to neuromyelitis optica, the cause of multiple sclerosis–a much more common disease in the western world—is undetermined despite many decades of intense research activity. It is thought to be T cell mediated and there are likely to be both genetic and environmental contributions [7]. The majority of multiple sclerosis patients develop a progressive phase either from onset, or more commonly secondary to a relapsing phase, and it is during this progressive phase that the majority of disability is incurred [8, 9]. A neurodegenerative process is thought to be the pathological substrate of this progression [10]. Understandably preventing neurodegeneration is the current focus of ongoing pharmaceutical research.

Imaging and pathological studies of multiple sclerosis have recognised that ‘normal appearing’ brain tissue on conventional imaging is not normal [11] and that subclinical activity with ongoing lesion formation and progressive atrophy occurs in clinically stable patients [12–14]. It is possible that the ‘unseen’ and chronic subclinical pathology, distinct from inflammatory lesions, may contribute to the neurodegenerative pathology in multiple sclerosis and progressive disability. An alternative explanation is that neurodegenerative changes occur as a result of chronic axonal demyelination secondary to inflammation [15, 16]. Notably, there is no progressive phase in neuromyelitis optica and the disability is solely relapse related [17, 18]. Thus studying neuromyelitis optica may help to determine which of these two scenarios is more likely. If previous attacks of severe demyelination are a cause of subsequent continuous neurodegeneration, then in neuromyelitis optica, where the demyelination is generally more severe than in multiple sclerosis [19, 20], one would expect ongoing tissue damage and non-relapse related progression. However if subclinical activity and non-lesional pathology is driving the progressive phase of multiple sclerosis then these features may be absent in neuromyelitis optica. To date, there are no reported prospective imaging studies in neuromyelitis optica and cross-sectional retrospective studies give varying results [21], perhaps due to the inclusion of seronegative neuromyelitis optica patients in several studies or the relative lack of sensitivity to change in cross-sectional compared to longitudinal analyses.

Studying the imaging features of neuromyelitis optica and contrasting them to multiple sclerosis is important for understanding the relationship between inflammation, demyelination and neurodegeneration. The primary objective of this study was to assess whether non-lesional pathology and chronic subclinical activity occurs in neuromyelitis optica as it does in multiple sclerosis using quantitative MRI. To address this aim we conducted a longitudinal MRI study of aquaporin-4 antibody positive neuromyelitis optica patients, relapsing remitting multiple sclerosis patients and healthy controls. We included quantitative MR acquisition methods: diffusion tensor imaging to measure neuronal and glial integrity [22], myelin water imaging to indicate myelin density and damage [23, 24], and magnetization transfer imaging of the spinal cord [25] to show areas of damage to the normal appearing tissue. To detect atrophy we used volumetric measures of brain, subcortical and cervical spinal cord volume that have become accepted surrogates of neurodegeneration [26, 27]. Subclinical lesion activity was assessed by comparing baseline and one year lesion loads, in the absence of relapses. In a follow up to our previous work in identifying conventional imaging features that separate neuromyelitis optica from multiple sclerosis [28] we explored whether these quantitative imaging measures can help to distinguish neuromyelitis optica and multiple sclerosis using discriminant analysis.

## Materials and Methods

### Ethics and design

The study was designed as a prospective longitudinal MRI study consisting of brain and cervical spinal cord imaging at baseline, and brain imaging at one year follow up. It was approved by the South East Hampshire NHS Research Ethics Committee (REC reference number 09/H0501/55), in accordance with the declaration of Helsinki.

### Subjects

Patients and controls over the age of 18 were eligible for this study. 18 neuromyelitis optica patients recruited from the NHS Neuromyelitis Optica Highly Specialized Service in Oxford, 15 relapsing remitting multiple sclerosis patients who fulfilled MacDonald’s revised criteria [29], and 17 healthy controls attended for the baseline cross-sectional study. All participants gave informed written consent to participate. 16 neuromyelitis optica patients, 13 multiple sclerosis patients and 16 healthy controls returned for a one year follow-up. All subjects were tested for the presence for serum aquaporin-4 antibodies using the cell-based assay method [6]. All neuromyelitis optica patients were positive, whereas the multiple sclerosis patients and controls were all negative. All multiple sclerosis patients had previously had two or more relapses disseminated in time and space.

### Imaging

The MRI brain scan was performed at 3 Telsa (Siemens Magnetom Verio, Erlangen, Germany) using a 32 channel receive head coil. Brain MRI included structural 3-dimensional T1 weighted scans for volumetric analysis with axial 2-dimensional T2, proton density and FLAIR imaging for lesions detection, 60 direction diffusion tensor imaging and myelin water imaging using the mcDESPOT multicomponent technique [30]. The cervical spinal cord MRI included structural T1 and T2 weighted sequences for volumetric analysis, and magnetization transfer weighted imaging. This full MRI protocol was repeated at one year.

Imaging sequences are given in detail in the supporting information (S1 Text).

### MRI Analysis

MRI analysis was carried out with the FMRIB Software Library (FSL version 4.1.9 [31]). Detailed explanations of the MRI analysis pipelines can be found in the supporting information (S1 Text).

### Baseline and 1 year Lesion load

T2 lesions were manually segmented from the baseline and one year FLAIR images, using the T2 and proton density volumes for reference. Lesion load (i.e. the total volume of lesions) was calculated using FSL, and the lesion number counted manually.

### Volumetrics

#### Whole brain volume

This was normalised for intra-cranial volume. Cross-sectional normalized brain volume and percentage volume change were analysed using the semi-automated software SIENAX and SIENA respectively [32].

#### Thalamic volume

This was calculated using the semi-automated segmentation tool FIRST [33]. Prior to segmentation the images were bias-field corrected and lesions filled as described by [34]. They were then normalized for intra-cranial volume.

#### Voxel-Based Morphometry

Group differences in regional brain volumes were also investigated using FSL-VBM, a voxel-based morphometry style analysis [35].

#### Cervical spinal cord volume of the normal appearing tissue

14 of the neuromyelitis optica patients and 10 of the multiple sclerosis patients had evidence of spinal cord lesions within the cervical spinal cord (the area of interest) at the time of the baseline scan, as visualized on the T2 weighted sagittal and axial images. All slices containing lesions were excluded for examination of the normal appearing spinal cord tissue. The superior cervical spinal cord was used for atrophy measurements due to better quality or image acquisition of this portion of the cord. During acquisition the first (or most superior) axial slice was positioned at the superior border of C2 (i.e. the odontoid process of the epistropheus or axis) using a sagittal T2 weighted image. The spinal cord was segmented using the Horsfield method [36] now incorporated into Jim 6.0 (Xinapse Systems). The volume of the spinal cord was measured over 11 axial slices of 3.3mm starting with the most superior slice. The options used were 32 shape coefficients to describe the complexity of the cord outline and an order of 12 polynomial variations of the shape coefficient along the cord.

### Quantitative Imaging of the Normal Appearing Tissue

#### Diffusion tensor imaging of the normal appearing white matter

Voxel-wise statistical analysis of the fractional anisotropy (FA) data was carried out using Tract-Based Spatial Statistics (TBSS; [37]).

A subgroup region of interest analysis was conducted using the mean derived TBSS skeleton for the neuromyelitis optica versus controls group analysis. A white matter atlas was used to manually define the region of interest within the optic radiations. The mean FA and standard deviation for each subject’s optic radiations were extracted for right and left separately excluding lesions. From the neuromyelitis optica group, two subgroups were selected; four patients with bilateral severe visual impairment (visual acuity ≤ 0.1) due to previous optic neuritis and six patients with good functional vision (visual acuity ≥6/9; n = 6).

#### Myelin water imaging of the normal appearing white matter

mcDESPOT processing was performed as described by [30] and [38] to form the myelin water fraction (MWF). This MWF data was aligned to standard space using non-linear registration and then projected onto a white matter skeleton using the projections that were derived from the TBSS analysis of FA described above.

#### Cervical cord magnetisation transfer contrast normalized with CSF signal (MTCSF) of the normal appearing tissue

The magnetization transfer weighted slices were acquired from the top of the superior border of C2, i.e. the superior border of the odontoid peg, positioned using a T2 weighted sagittal image. The most superior six slices, each of 4mm thickness, were examined which covered approximately the C1-C2 spinal cord segment. Each slice was normalized by dividing the intensity values by the mean intensity value of the CSF in a region of interest taken from the non-magnetisation transfer weighted volume of the same slice. Other regions of interest were taken in the normal appearing grey matter and white matter. Whole slices containing lesioned tissue were excluded. To quantify loss of tissue integrity (which would be seen visually as a lack of contrast between white and grey matter) a ratio of white:grey matter normalized contrast was calculated for each patient, in each separate slice from C1-C6, and averaged across each group.

### Statistics

The group average brain, thalamic, spinal cord volumes and MTCSF white:grey matter ratios, FA and MWF and optic radiation FA were compared with analyses of co-variance (ANCOVA) with age (and time between scans for the longitudinal data) as co-variates, with a Tukey test to indentify the pairwise differences between the three groups (controls, multiple sclerosis and neuromyelitis optica).

Longitudinal changes in brain volume within each group, measured using the SIENA software, were also tested with a voxelwise analysis that identifies atrophy or growth and the location of the change [32].

FA and MWF data were compared voxel-wise according to group using permutation-based non-parametric testing, and corrected for multiple comparisons. Lesion maps were entered as a nuisance regressor in the final model i.e. lesions were excluded. For the longitudinal data a mid-space template, was used to register both sets of images.

A paired comparison of baseline and one year lesion load was made using Wilcoxon sign-rank test.

#### Discriminant Analysis

To explore whether it is possible to find a method of distinguishing neuromyelitis optica and multiple sclerosis using the measures derived above, a discriminant analysis was performed using the quantitative imaging measures derived. A discriminant analysis is a method akin to regression that is used for classification when the dependent variables are categorical, and the predictor variables are at interval level. The analysis was automated and run using SPSS version 19. The outcome variable was group (i.e. MS or neuromyelitis optica), and all cross-sectional and longitudinal predictor variables were included. A stepwise approach was used where the derivation was simplified by disregarding variables that highly correlate with the variable that explains the greatest variance.

## Results

Subject characteristics are given in Table 1. There was good matching between sex and disease duration was obtained. However, multiple sclerosis patients were younger than the controls and neuromyelitis optica patients, therefore all between groups comparison are corrected for age. The disability of the neuromyelitis optica patients was higher due to previous severe transverse myelitis and/or optic neuritis. Neuromyelitis optica patients were receiving immunosuppressant medication and active relapsing multiple sclerosis patients were on disease modifying therapy.

**Table 1:** Subject Characteristics.

Subject Group	NMO	MS	Control
Number attending baseline	18	15	17
Age, Range (median)	20–76 (46)	22–62 (38)	21–77 (47)
Sex	15F, 3M	11F, 4M	13F, 4M
Aquaporin-4 antibody positive	18	0	0
EDSS, range (median)	2–6 (4)	0–5 (2)	-
Disease Duration, range (median)	12–186 (57.5)	24–240 (72)	-
Number Attending Follow-up at 1 year	16	13	16
Age attending follow-up, range (median)	20–76 (46)	29–62 (40)	21–77 (47)
Number of days to follow-up, range (median)	350–456 (386)	346–420 (378)	340–430 (372)
Immunomodulation	Aza 9, Mtx 2, Pred 1, Aza+Pred 3, Mtx+Pred 3	Inf 3, Glt 6, None 6	-
Number with brain lesions at baseline MRI	13	15	-
Number with cervical spinal cord lesion at baseline MRI	14	10	-
Number with severe visual impairment (VA ≤ 0.1).	9 (4 of which had bilateral severe visual impairment)	0	-

Abbreviations:

Aza: Azathioprine

Glt: Glatiramer

Inf: β-Interferon

Mtx: Methotrexate

MS: Multiple sclerosis

NMO: Neuromyelitis optica

Pred: Prednisolone

VA: Visual Acuity.

### Longitudinal Lesion Load

No patients relapsed in the one year follow up, probably related to their immunosuppressive/immunomodulatory therapy. Within the neuromyelitis optica cohort baseline (2049.7 mm^3^) and one year (1929.7 mm^3^) mean lesion load did not differ significantly. There was no change in the number of lesions for the whole neuromyelitis optica group. In comparison there was a significant difference in mean baseline (8648.8 mm^3^) and one year (9581.3 mm^3^) lesion load (p = 0.021) in the MS cohort, with an average change of +10.6%. Correlation analysis between the change in lesion load and change in normal appearing white matter FA over one year was conducted in the MS group showing no correlation (Pearson’s correlation coefficient of 0.293).

### Cross-sectional Quantitative Imaging

The results of the quantitative imaging studies are summarised in Table 2.

**Table 2:** Group comparisons of quantitative imaging measures.

	Group mean values (SD)				Pairwise Comparisons (performed when ANCOVA showed between groups difference).		
	Control	MS	NMO	ANCOVA F Score (p)	NMO vs Control	MS vs Control	NMO vs MS
Cross-sectional normalized brain volume/ mm^3^	1431785 (75185)	1432448 (82061)	1440255 (66069)	0.269 (0.765)	NS	NS	NS
Cross-sectional average thalamic volume/ mm^3^	9864.9 (602.5)	8749.6 (866.1)	9991.4 (552.7)	**10.34 (<0.001)** *	NS	**p<0.001** *	**p<0.001** *
Cross-sectional cervical spinal cord volume of NAT/ mm^3^	2945.7 (148.5)	2684.3 (252.3)	2848.7 (131.8)	**5.48 (0.003)** *	NS	**p = 0.001** *	**p = 0.041** *
DTI of NAWM (FA)	0.473 (0.015)	0.443 (0.029)	0.454 (0.027)	**5.021 (0.011)** *	NS	**p = 0.010** *	NS
MWI of NAWM (MWF)	0.239 (0.005)	0.221 (0.009)	0.238 (0.008)	**4.639 (0.014)** *	NS	**p = 0.013** *	NS
Cervical cord MTCSF white:grey ratio of NAT/ mm3	0.847 (0.029)	0.887 (0.052)	0.833 (0.046)	**0.033 (0.01)** *	NS	**p = 0.023** *	**p<0.001** *
Percentage change in brain volume over one year / mm3	0.068 (1.078)	-0.526 (1.135)	-0.222 (1.507)	1.089 (0.38)	-	-	-
Change in average thalamic volume over one year / mm^3^	75.34 (143.1)	-136.24 (127.5)	158.54 (238.4)	**9.272 (<0.01)** *	NS	**p = 0.007** *	**p<0.001** *

Abbreviations:

ANCOVA: analysis of co-variance

DTI: diffusion tensor imaging

MTSCF: Magnetisation transfer contrast normalized by CSF signal

MWI: myelin water imaging

NAT: Normal appearing tissue

NAWM: normal appearing white matter

NS: not significant

SD: standard deviation

*: significant difference (p<0.05).

#### Volumetrics

**Fig 1:**
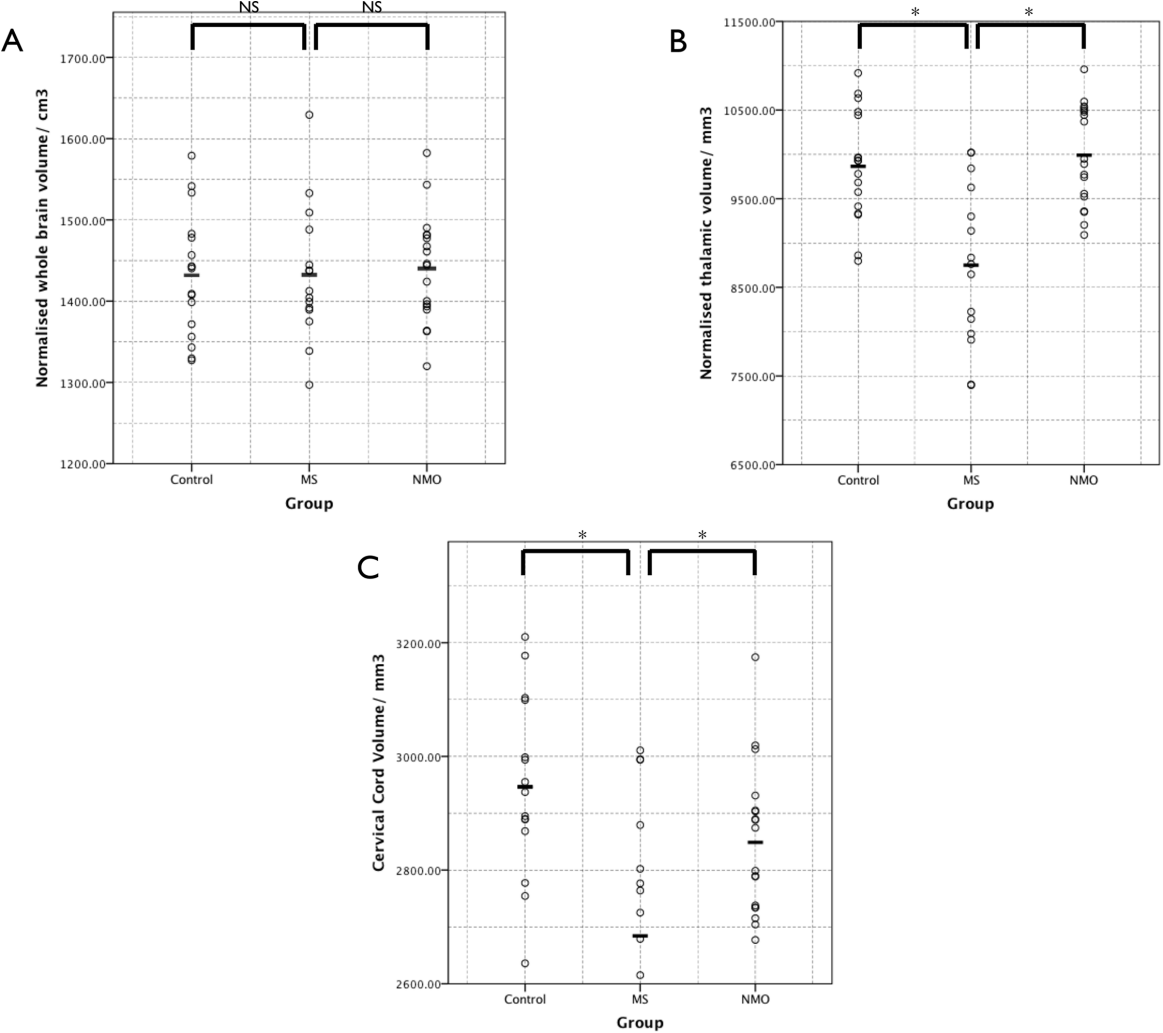
Results of the cross-sectional volumetric analyses. The dots show individual patient results and the bars the group mean. Individual and mean results have not been normalised for age. (A) Whole brain volume normalised for intracranial volume. (B) Thalamic volume normalised for intracranial volume. (C) Cervical spinal cord volume. NS = not significant, * = significant difference (corrected p< 0.05).

There were no significant differences between baseline whole brain volume between the three study groups (Fig 1A). However average thalamic volume was significantly lower in multiple sclerosis patients when compared to both neuromyelitis optica and controls (Fig 1B). There was no significant difference between neuromyelitis optica and controls.

Normal appearing tissue mean spinal cord volumes for the C1 and C2 segments were significantly lower in the multiple sclerosis group than both the control and neuromyelitis optica groups (Fig 1C).

**Fig 2:**
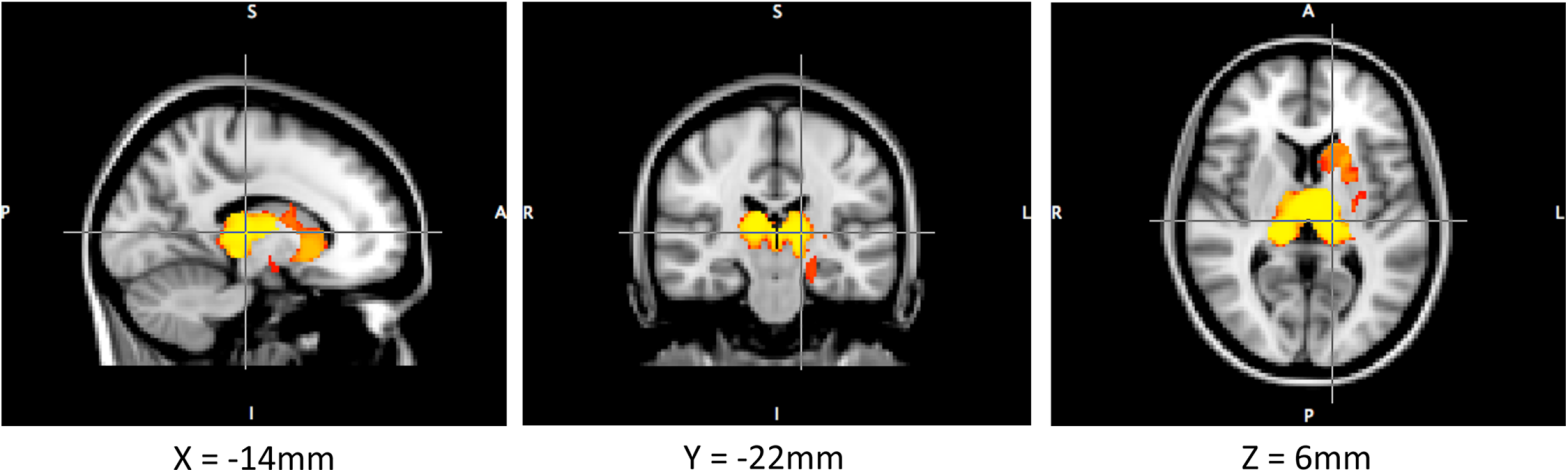
Voxel-based morphometry of the multiple sclerosis group compared to controls. Showed relative atrophy of the thalami and caudate nuclei.

Voxel-based morphometry of the cross-sectional data comparing multiple sclerosis with controls also showed prominent grey matter volume loss in the thalami of multiple sclerosis patients (Fig 2) as well as in the region of the left caudate nucleus. There was no significantly different area of grey matter volume between the controls and neuromyelitis optica patients.

#### Diffusion tensor imaging of the normal appearing white matter

**Fig 3:**
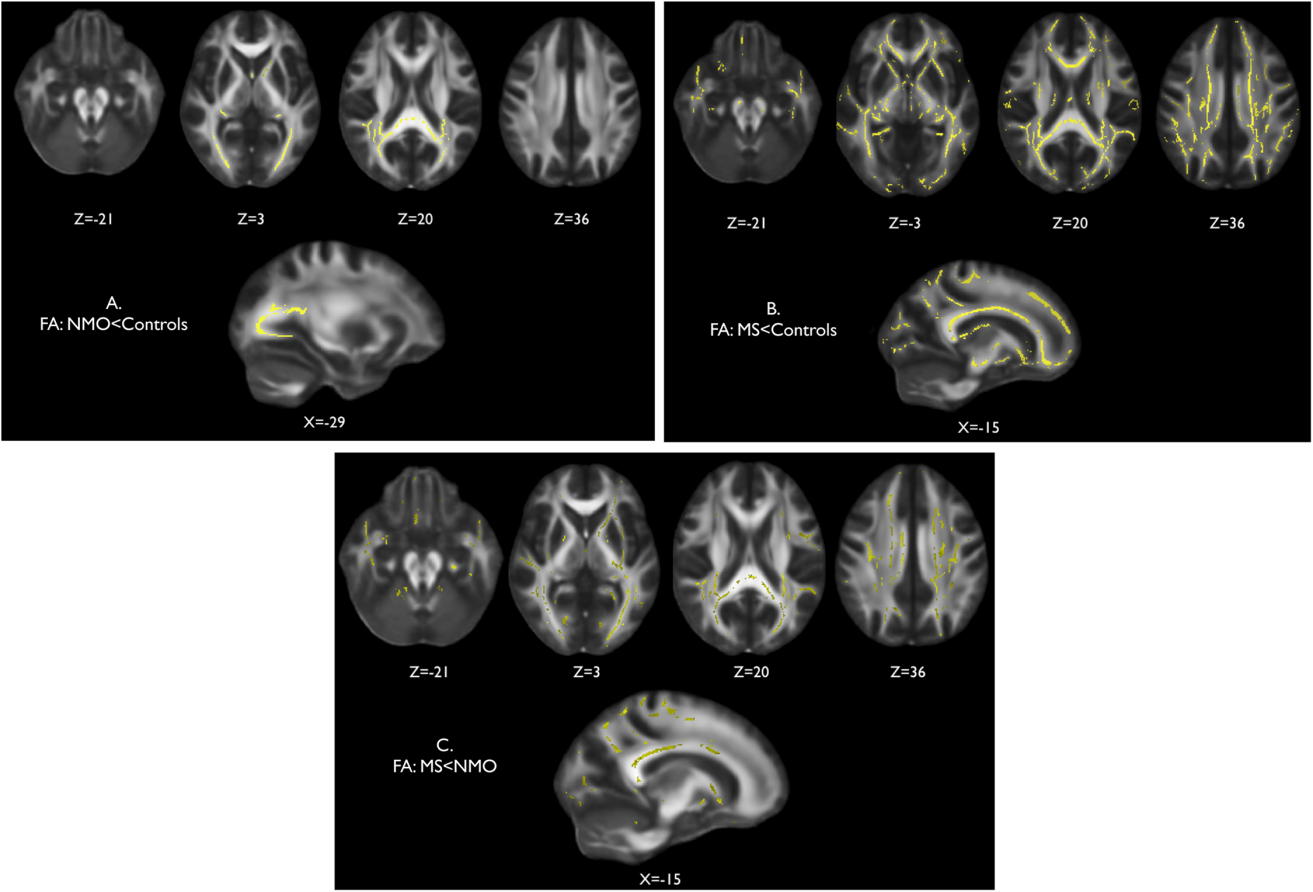
Cross-sectional fractional anisotropy of the normal appearing white matter. Voxelwise comparison of fractional anisotropy (FA) within white matter skeletons created with TBSS where significantly lower FA is shown in yellow for (A) neuromyelitis optica group versus control group, (B) multiple sclerosis group versus control group, and (C) multiple sclerosis group versus neuromyelitis optica group.

Tract-wise comparison of normal appearing white matter FA between neuromyelitis optica patients and controls, and multiple sclerosis patients and controls, showed notable differences in the location of abnormalities in each condition. Neuromyelitis optica is associated with a significantly reduced FA in the normal appearing white matter of the optic radiations (Fig 3A). There are also some significant FA reductions in parts of the splenium of the corpus callosum and the posterior corona radiata. Multiple sclerosis is associated with widespread changes throughout the normal appearing white matter (Fig 3B) when compared to controls. There were also widespread significant reductions in the FA of the multiple sclerosis cohort when compared to neuromyelitis optica (Fig 3C). Significant differences were observed throughout the supra and infratentorial white matter in regions including the superior longitudinal fasciculus, corpus callosum, corona radiata, internal and external capsule, inferior longitudinal fasciculus and cerebellum. Conversely, no areas of significant FA reduction were found in the neuromyelitis optica cohort when compared to multiple sclerosis.

Region of interest analysis comparing the mean FA within the optic radiations of a subgroup of neuromyelitis optica patients with bilateral severe visual impairment (visual acuity ≤ 0.1) and a subgroup of neuromyelitis optica patients with good functional vision and healthy controls showed significantly lower FA in the patients with bilateral blindness only (p<0.05). This suggests that the FA reduction found within the optic radiations in the neuromyelitis optica group when compared to controls is driven by a subset of patients with severe visual impairment. As there were no multiple sclerosis patients with severe chronic visual impairment, and the white matter changes were more diffuse in the multiple sclerosis group, there was no relationship between previous optic neuritis and a reduction of FA within the optic radiations.

The mean FA of normal appearing white matter was significantly lower in the multiple sclerosis compared to control group. Taken as a whole, there was no significant difference between the FA of normal appearing white matter of the multiple sclerosis and neuromyelitis optica groups, however removing the four neuromyelitis optica patients with severe bilateral visual impairment results in a statistical difference (FA, neuromyelitis optica>multiple sclerosis, p = 0.043).

#### Myelin water imaging of the normal appearing white matter

**Fig 4:**
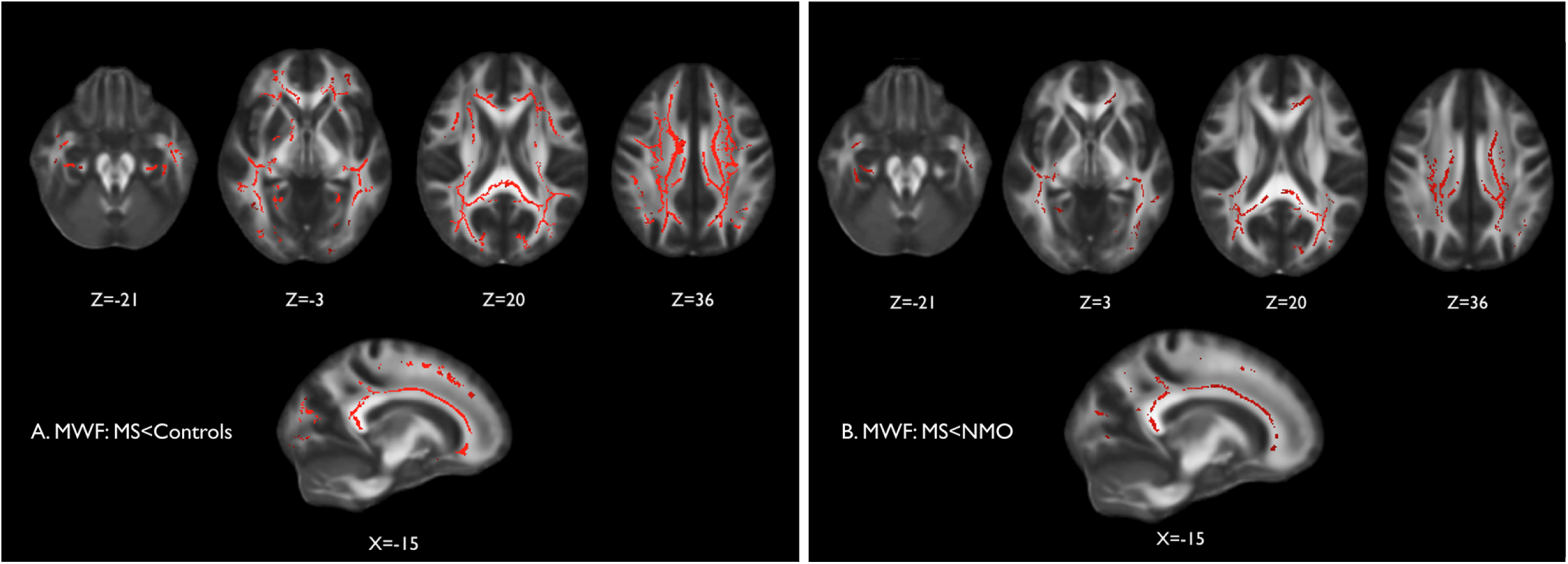
Cross-sectional myelin water fraction (MWF) of the normal appearing white matter. Voxelwise analysis of MWF in the white matter skeleton created with TBSS where significantly lower MWF is shown in red for (A) multiple sclerosis group versus control group, and (B) multiple sclerosis group MWF versus neuromyelitis optica group.

Tract-wise analysis of the normal appearing white matter myelin water fraction in multiple sclerosis and controls was consistent with findings from diffusion-weighted imaging, with widespread significant reductions in the multiple sclerosis group (corrected p<0.05) relative to controls (Fig 4A) and to neuromyelitis optica (Fig 4B). There was no significant difference in MWF between neuromyelitis optica and controls.

The mean MWF in the normal appearing tissue is significantly lower in the multiple sclerosis compared to control group. There was no significant difference between the multiple sclerosis and neuromyelitis optica groups.

#### Cervical cord magnetisation transfer contrast normalized with CSF signal (MTCSF) of the normal appearing tissue

**Fig 5:**
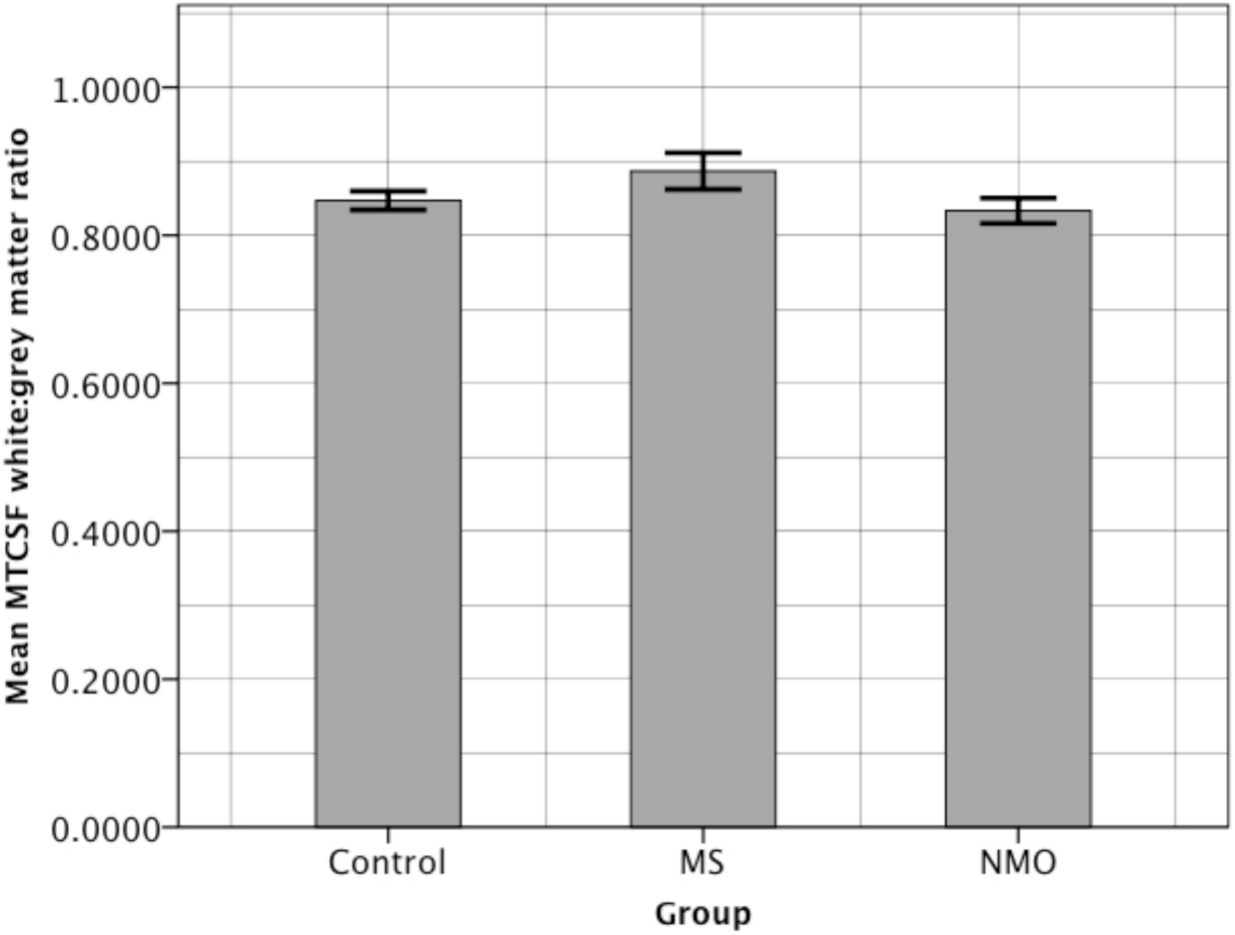
Bar graph showing average spinal cord MTCSF white:grey matter ratio in each patient group. Error bars are +/- 2 standard errors of the mean.

There is a significantly higher white:grey matter ratio (indicating a loss of spinal architecture) in the multple sclerosis group compared with the control and neuromyelitis optica groups (p<0.05; Fig 5). There were no significant differences in the normal appearing tissue of neuromyelitis optica patients when compared to controls.

### Longitudinal Quantitative Imaging

#### Volumetrics

**Fig 6:**
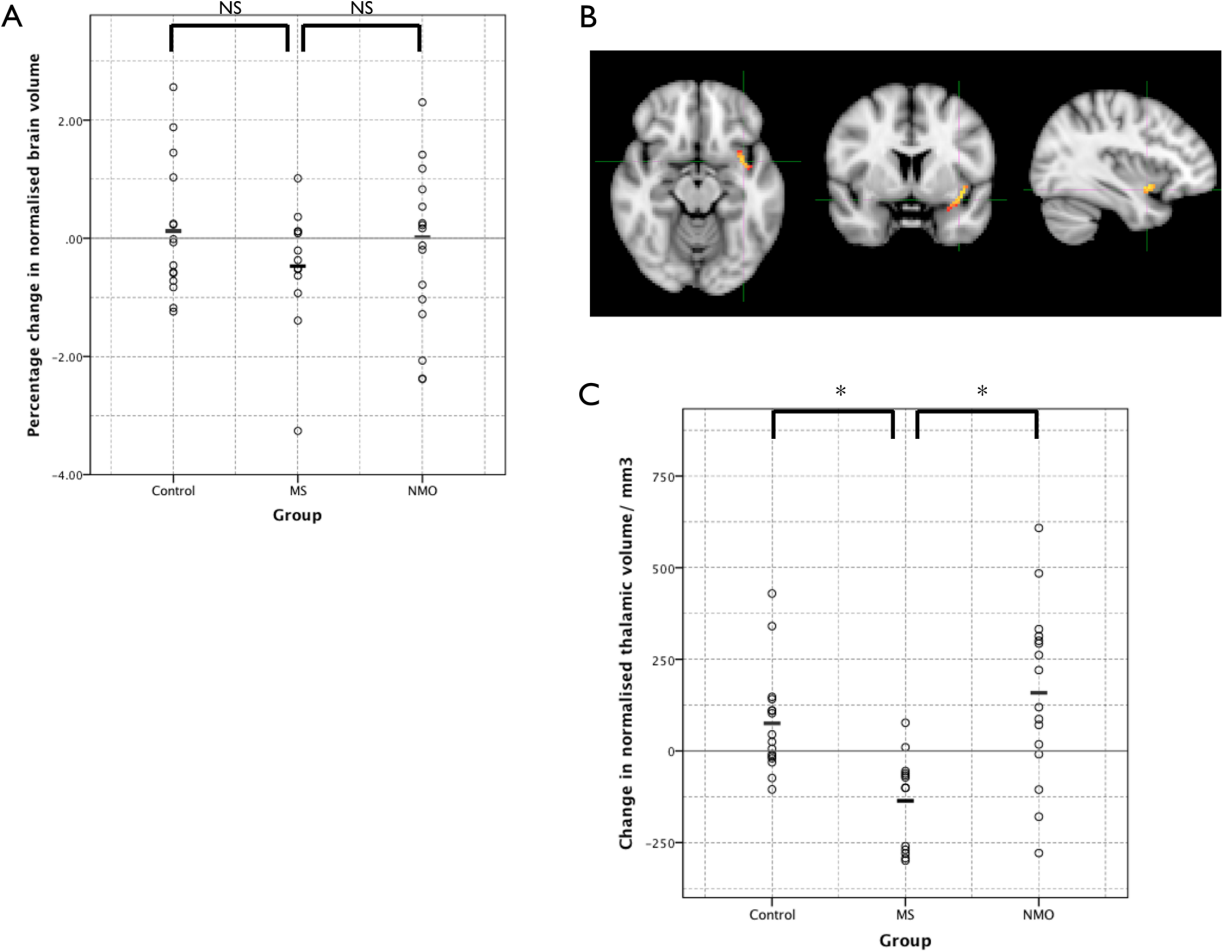
Longitudinal volumetric measures. (A) Percentage change in brain volume over one year in subject groups. (B) Voxelwise within-group analysis found a small significant area of atrophy in the region of the insula cortex within the multiple sclerosis group (shown) but not the neuromyelitis optica or control groups over the course of one year. (C) Change in thalamic volume over one year. NS = not significant, * = significant difference (corrected p< 0.05).

The average percentage change in brain volume over one year in each subject group is shown in Fig 6A. It is greatest in the multiple sclerosis group, and least in the control group but ANCOVA statistical testing with age as a co-variate found there was no significant difference between groups (between groups effect is p = 0.375). However a voxel wise examination (using the tool SIENAr) of whether there were any significant areas of whole brain atrophy within the group showed a small area in the region of the left insula cortex in the multiple sclerosis group (corrected P <0.05; Fig 6B) but no significant areas within the control and neuromyelitis optica groups.

Repeated measures analysis of thalamic volume at baseline and one year showed a significant decrease in the multiple sclerosis group compared to both the neuromyelitis optica and control groups (p<0.01). Change in thalamic volume in each group (unadjusted for age) is shown in Fig 6C.

#### Diffusion tensor imaging of the normal appearing white matter

**Fig 7:**
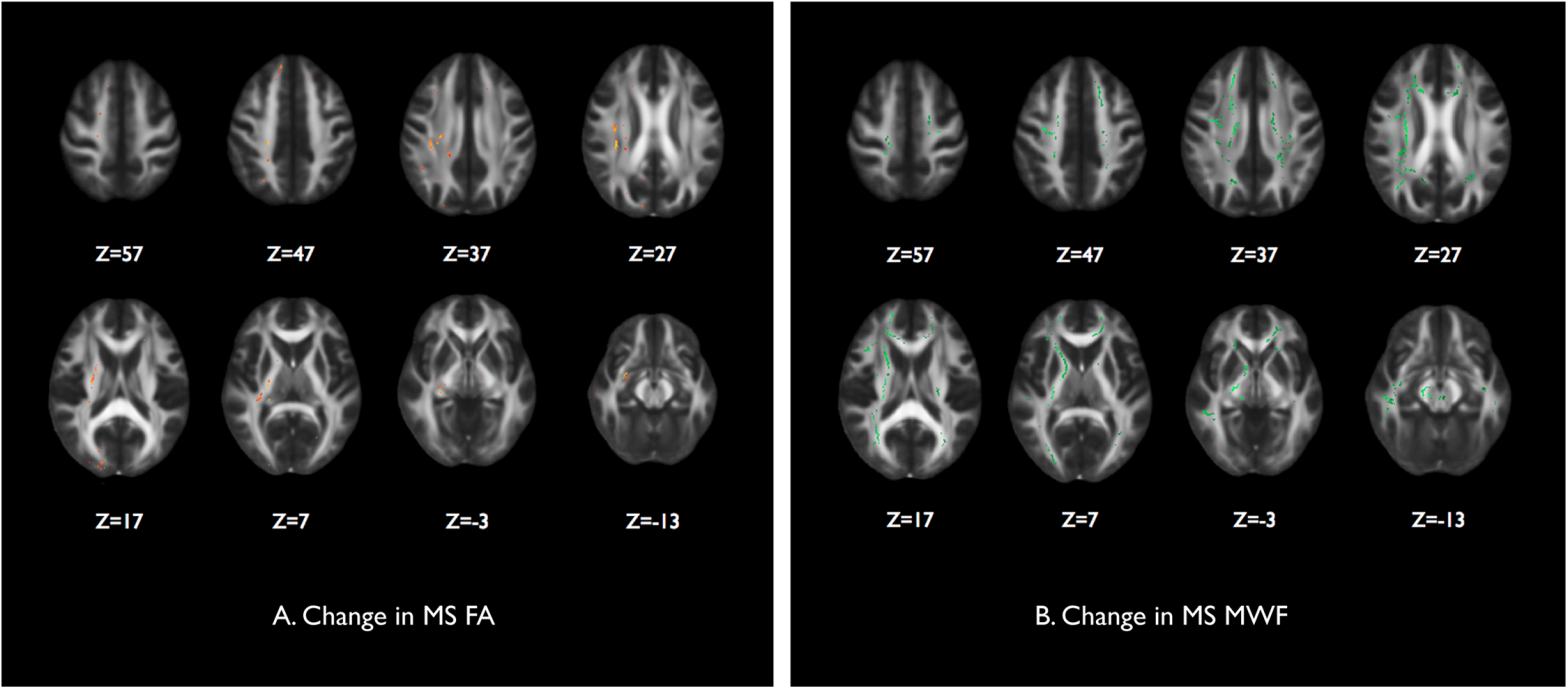
Longitudinal measures of the integrity of the normal appearing white matter. Voxelwise paired comparison of baseline and one year scans for each subject group showed significant areas within the TBSS white matter skeletons of the multiple sclerosis group of (A) reduction in fractional anisotropy shown in red/yellow, and (B) reduction in myelin water fraction shown in green.

There were no significant changes in the normal appearing white matter FA between the baseline and one year scans in the control and neuromyelitis optica groups. In the multiple sclerosis group there were scattered areas of significant reduction in FA throughout the normal appearing white matter as shown in Fig 7A. These regions include the right uncinate fasciculus, right corticospinal tract, right internal capsule, right inferior longitudinal fasciculus, left anterior thalamic radiation, right corona radiata, right juxtacortical white matter. Other left hand sided changes were also seen in a similar distribution to the right that were just below the significance threshold (i.e. a trend).

#### Myelin water imaging of the normal appearing white matter

There were no significant changes between baseline and 1 year scans in the control and neuromyelitis optica groups. As displayed in Fig 7B there are some areas of the significant reduction in MWF in the normal appearing white matter of the multiple sclerosis group. These are in the regions of the left and right juxtacortical white matter, the left and right corticospinal tracts, left and right internal capsule, the body and genu of the corpus callosum, the right anterior and posterior thalamic radiations, the right optic radiation, the left and right cerebral peduncles and inferior longitudinal fasciculi.

### Discriminant Analysis

The optimal discriminant function derived was:

D=−0.01NWBV+2ATV−0.891

where NWBV = cross-sectional normalised whole brain volume in cm^3^ and ATV = cross-sectional average (of left and right) thalamic volume in cm^3^.

**Fig 8:**
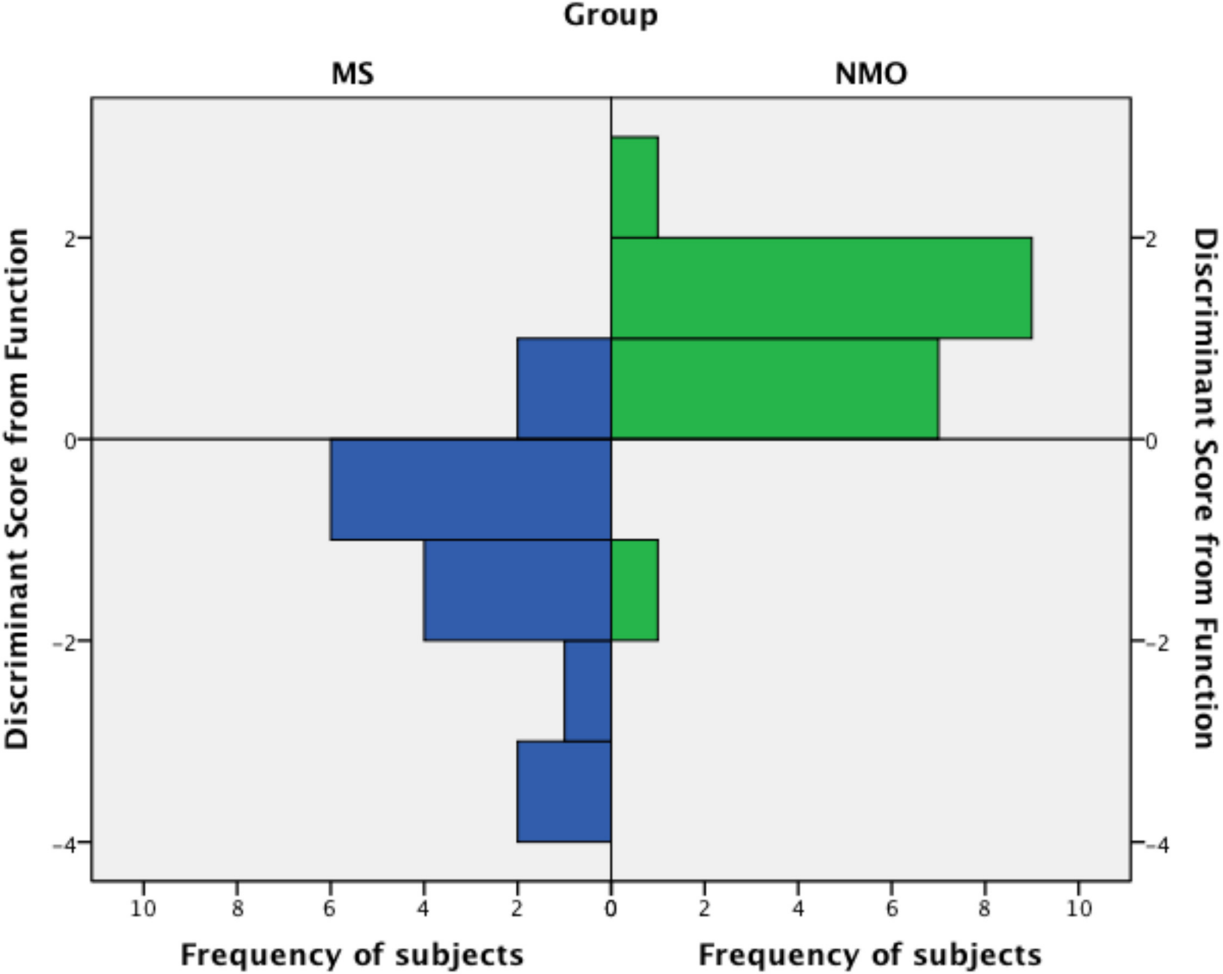
Histogram showing the frequency of subjects (x axis) classified into each group by their discriminant function score (y axis).

Neuromyelitis optica cases would be expected to centre around 0.983 (standard deviation 0.86) and multiple sclerosis around -1.114 (standard deviation 1.22). 0.891 is a constant to allow zero to be the dividing line for the groups. It is able to correctly classify 90.9% of subjects when cross-validated (Table 3; Fig 8). This equates to 89.5% sensitivity, 92.9% specificity, 94.4% positive predictive value and 86.7% negative predictive value for the diagnosis of neuromyelitis optica.

**Table 3:** Cross-validation of function for the separation of neuromyelitis optica and multiple sclerosis.

		Predicted Group Membership		
		MS	NMO	Total
Original Group Membership	MS	13	2	15
	NMO	1	17	18

## Discussion

The consistent outcome of this study is that widespread neurodegenerative changes can be demonstrated in the multiple sclerosis group, but not the neuromyelitis optica group, for both the cross-sectional quantitative measures (thalamic volume, normal appearing white matter FA and MWF, cervical spinal cord volume and MTCSF) and the longitudinal results (temporal brain atrophy, thalamic atrophy, normal appearing white matter FA and MWF). More localised changes have been noted in the neuromyelitis optica group with decreased FA in the optic pathways that appear to be driven by the inclusion of patients who are blind or have severe visual impairment. A further finding of this study is that in the absence of relapse lesion load remains static in the neuromyelitis optica group. Together with the lack of progressive neural damage in the normal appearing tissue over the one year period, this suggests that there is no detectable disease activity in neuromyelitis optica between relapses.

Both multiple sclerosis and neuromyelitis optica involve inflammatory brain lesions, but the diffuse neurodegenerative changes have only been found in multiple sclerosis. In support of this finding is the clinical observation that progression is very rare or absent in neuromyelitis optica [17, 18] whereas it occurs in the majority of multiple sclerosis patients, either as a primary phenomenon or secondary to a relapsing remitting phase. Furthermore to date there are no published pathological studies that show axonal or myelin loss in the normal appearing tissue of neuromyelitis optica patients. The inflammatory brain and spinal cord lesions in neuromyelitis optica tend to be pathologically more destructive than those in multiple sclerosis [19], therefore the findings of our study cast doubt on the theory that the chronic neurodegenerative changes found in multiple sclerosis are a secondary downstream/ upstream effect of active demyelinating lesions. Further supporting this, the reduction in normal appearing white matter FA in the multiple sclerosis group did not correlate with the increase in lesion load. Instead either a more diffuse inflammatory process [39] or two separate disease mechanisms (i.e. inflammatory and neurodegenerative) could be responsible [40].

This study also demonstrates the greater sensitivity of thalamic volume over whole brain volume as a surrogate of neurodegeneration in small numbers of relapsing remitting multiple sclerosis patients. Additionally we show diffusion tensor imaging and myelin water imaging were more sensitive than whole brain atrophy in detecting changes over a year in a small multiple sclerosis cohort, and that these changes were not present in neuromyelitis optica patients studied over the same period. These methods may therefore be of use diagnostically, and in clinical treatment trials of potential neuroprotective agents for multiple sclerosis.

A further outcome of this study is the derivation of a formula that utilises cross-sectional normalized brain and thalamic volume to aid the separation of neuromyelitis optica and multiple sclerosis with a high level of accuracy (89.5% sensitivity, 92.9% specificity). Fortuitously from a translational point of view these volumetric measure can be calculated using semi-automated methods [32, 33], and therefore, if validated, there is potential to bring this into clinical practice in the future.

Our earlier work in this area has shown that T2 lesion distribution on conventional MRI can also be used to separate a cohort of 50 multiple sclerosis patients from 44 seropositive neuromyelitis optica patients with a 92% sensitivity and 96% specificity [28]. The two multiple sclerosis patients in the present study that were misclassified as neuromyelitis optica by our new discriminant function both had Dawson’s finger type lesions and therefore would have satisfied the conventional imaging criteria for multiple sclerosis. Thus the combination of these validation methods may give greater accuracy when distinguishing multiple sclerosis from neuromyelitis optica. It should be noted that specificity and sensitivity values are relevant to the mix of the population tested. If our discriminating algorithm was applied to a group of patients with a larger proportion of multiple sclerosis patients then the specificity would be reduced. We have assumed that these algorithms will be applied to a cohort of patients where an experienced neurologist has already screened out conventional multiple sclerosis patients.

Previous literature in the field of quantitative imaging and neuromyelitis optica is inconsistent [41]. Whilst there does seem to be greater evidence supporting the notion that widespread neurodegeneration is absent in neuromyelitis optica [42–47], others have reported more diffuse changes [48, 49]. With respect to previous DTI studies of neuromyelitis optica, some have found localised differences in DTI indices in the optic radiations that corroborate with our work [43–46], with the addition of changes within the cerebral coriticospinal tracts [43]. More recent research has reported widespread DTI abnormalities [48, 49]. Similarly the results of volumetric work range from no apparent changes [50], to differing anatomical regions of both grey matter atrophy [51, 52] and white matter atrophy [53, 54]. Possible explanations for these differences include population demographics including race. In addition the potential inclusion of seronegative patients in studies of neuromyelitis optica introduces the risk that participants may have an opticospinal form of multiple sclerosis, misclassified as neuromyelitis optica. A further potential reason could be due to the precise methods used to exclude lesions from quantitative analysis of normal appearing white matter to avoid contamination.

There are limitations of this study. Although reductions in FA, MWF and basal ganglia volumes have been found in multiple sclerosis over a one-year interval [55, 56], a potential limitation of this work is that the follow-up period may not have been long enough to detect neurodegenerative changes in neuromyelitis optica. Additionally the sample size of multiple sclerosis and neuromyelitis optica groups were small, and a small effect in neuromyelitis optica (less than in multiple sclerosis) cannot be excluded – 280 relapsing remitting multiple sclerosis patients are required to detect a 30% treatment effect on whole brain atrophy [57], therefore it is not surprising that changes in whole brain volume, compared to controls over one year, were not detected in our multiple sclerosis cohort. Clearly the rarity of neuromyelitis optica, and our stipulation to only include patients antibody tested with the cell-based assay method to ensure a population with definite disease, makes a larger study challenging. Quantitative imaging is a surrogate of neurodegeneration and does not prove the pathological process, however it is currently our best method of studying the activity of neurological diseases non-invasively in-vivo. Despite these limitations our study does support the concept that there are differences in background disease activity and non-lesional pathology between neuromyelitis optica and multiple sclerosis.

Our study is the most comprehensive advanced neuroimaging study of neuromyelitis optica and multiple sclerosis performed to date, and is reassuring evidence that whilst neuromyelitis optica is clinically silent there appears to be no ongoing disease activity. It also further stimulates the debate on the cause of neurodegeneration in multiple sclerosis that is the biggest contributor to long-term disability, and for which we still have no effective treatments. Future work may include using quantitative MRI to study neuromyelitis optica lesions in evolution to try to reveal more about the mechanisms of astrocyte and myelin damage. Validation of our work in independent cohorts would also be valuable.

## Supporting Information

S1 Dataset Minimum dataset for study. Includes demographic details for patients and quantitative imaging data.
(XLSX)

S1 Text Supporting information regarding methodology. Includes 1. Demographic details of individual patients 2. MRI sequence parameters. 3. Further details of the MRI analysis pipelines.
(DOCX)
