# Supplementary material for: Imaging Surrogates of Disease Activity in Neuromyelitis Optica Allow Distinction from Multiple Sclerosis
Source: PLoS One. 2015 Sep 18;10(9):e0137715. doi: 10.1371/journal.pone.0137715 (PMC4575169; doi:10.1371/journal.pone.0137715)
Supplement: S1 Text — Includes 1. Demographic details of individual patients 2. MRI sequence parameters. 3. Further details of the MRI analysis pipelines. (DOCX) [file pone.0137715.s002.docx]

Supplementary Methods 1:

*Details of Individual Patients:*

*Neuromyelitis optica*

|  | Age* | Sex | Aquaporin-4 antibody status | Disease Duration/ months* | Previous Relapses** | Hand Dominance | Baseline EDSS | 1 year EDSS |
| --- | --- | --- | --- | --- | --- | --- | --- | --- |
| 1 | 50 | F | Positive | 50 | R+L ON | R | 3.5 | 3.5 |
| 2 | 42 | F | Positive | 24 | R ON, LETM | R | 3.5 | 3.5 |
| 3 | 28 | F | Positive | 186 | R+L ON | R | 4.0 | 4.0 |
| 4 | 33 | F | Positive | 69 | R+L ON, LETM, B | R | 5.5 | 6.0 |
| 5 | 56 | F | Positive | 60 | R+L ON, LETM | R | 6.0 | 6.0 |
| 6 | 20 | F | Positive | 84 | R+L ON, LETM | R | 3.5 | 3.5 |
| 7 | 33 | M | Positive | 48 | R+L ON, LETM | R | 4.0 | 4.0 |
| 8 | 76 | F | Positive | 180 | LETM | R | 7.5 | 6.5 |
| 9 | 46 | M | Positive | 183 | R+L ON, LETM | R | 4.0 | 4.0 |
| 10 | 40 | F | Positive | 74 | R+L ON, LETM | R | 3.5 | 3.5 |
| 11 | 63 | F | Positive | 24 | R+L ON | R | 2.0 | 2.0 |
| 12 | 70 | F | Positive | 55 | LETM | R | 6.0 | Did not attend |
| 13 | 66 | F | Positive | 72 | LETM | R | 6.0 | 6.0 |
| 14 | 60 | F | Positive | 60 | LETM | R | 3.0 | 2.0 |
| 15 | 40 | M | Positive | 36 | LETM | R | 5.0 | 4.5 |
| 16 | 38 | F | Positive | 12 | LETM, ON, B | R | 6.0 | Did not attend |
| 17 | 50 | F | Positive | 48 | LETM, B | R | 6.0 | 6.0 |
| 18 | 46 | F | Positive | 16 | ON, B, LETM | R | 4.0 | 4.5 |

*Multiple Sclerosis*

|  | Age* | Sex | Disease Duration/ months* | Previous Relapses** | Hand Dominance | Baseline EDSS | 1 year EDSS |
| --- | --- | --- | --- | --- | --- | --- | --- |
| 1 | 61 | M | 228 | R+L ON, TM, B | L | 5 | 5.5 |
| 2 | 49 | F | 54 | ON, TM, B | R | 1.5 | 1.5 |
| 3 | 30 | F | 120 | L ON, TM, B | R | 1.0 | Did not attend |
| 4 | 56 | F | 72 | R ON, TM, B | R | 5 | 4.5 |
| 5 | 29 | F | 36 | TM, B | R | 2 | 1.5 |
| 6 | 34 | M | 48 | TM, ON, B | R | 2 | 2.5 |
| 7 | 38 | F | 156 | TM, B | R | 1 | 1 |
| 8 | 40 | M | 72 | TM, B | L | 3.5 | 4.0 |
| 9 | 36 | M | 80 | ON, TM, B | R | 5 | 5 |
| 10 | 33 | F | 50 | ON, TM, B | L | 5 | 5 |
| 11 | 22 | F | 24 | ON, B | R | 0 | Did not attend |
| 12 | 34 | F | 26 | ON, TM. B | R | 2 | 1.5 |
| 13 | 60 | F | 240 | TM, B | R | 5 | 5 |
| 14 | 62 | F | 156 | R ON, B | R | 2 | 2 |
| 15 | 47 | F | 36 | B, TM | R | 4 | 4 |

Supplementary Methods 2: MRI Sequence parameters:

-Structural Imaging: 3 dimensional MPRAG sequence with voxel size 1x1x1mm, TR 2040ms, TE 4.7ms, 1 average.

-Sequences for Lesion Detection: T2: 2 dimensional turbo spin echo, 48 slices, slice thickness 3mm, voxel size 0.5x0.5mm, FOV 220x220mm, TR 5690ms, TE 71ms, 1 average. Flair: 2 dimensional turbo spin echo 48 slices, slice thickness 3mm, TE 73.0ms, TR 9000ms, voxel size 0.9x0.9. 1 average. PD: 2 dimensional turbo spin echo, 48 slices, slice thickness 3mm, voxel size 0.5x0.5mm, FOV 220x220mm, TR 3000ms, TE 18ms, 1 average.

-DTI: 60 diffusion directions with B-value 1000 and 8 volumes with no diffusion weighting, TR 9600ms, TE 87 ms, FOV 190 x 190mm, voxel-size 2.0x2.0x2.0mm, 65 slices, 1 average. Acquisition time = 12mins.

-MWI: Acquired with mcDESPOT (1). FOV=22x22x16cm, 1.7mm isotropic resolution; SPGR: TE/TR = 2.5ms/5.6ms, α={3,4,5,6,7,9,13,18}°, BW=±24kHz; SSFP: TE/TR=2.2ms/4.4ms, α={10,13.3,16.6,20,23.3,30,43.2,60}°, BW=±36kHz, phase-cycling patterns = 0º and 180º (for correction of off-resonance effects). A reduced resolution IR-SPGR image was also acquired with TE/TR/TI/α = 2.5ms/5.6ms/450ms/5° to correct for flip angle inhomogeneity. Acquisition time = 14mins.

-Spinal cord structural imaging: Both a 3-dimensional T1 and an axial Siemen’s MEDIC (a multi echo gradient echo sequence with T2 contrast) sequences were piloted with the spinal cord segmentation software. Segmentation was found to be more accurate with the MEDIC. This sequence was acquired with 30 consecutive axial slices with a distance factor of 10% of 3mm slice thickness. In plane voxel size 0.7x0.7mm. TE 17ms, TR 470ms. 2 averages. The most superior slice was positioned at the superior border of C2 (i.e. the odontoid peg). In order to localize lesions and position the axial slices a sagittal T2 turbo spin echo sequences were acquired. Sagittal: 15 slices of 3mm, voxel size 0.6x0.6, TE 67ms, TR 4590ms.

MTCSF: 2-dimensional gradient-echo sequence with twenty consecutive axial slices with a distance factor of 10% positioned at the superior border of the C2 vertebra. FOV 180mm x180mm, slice thickness 4mm, voxel-size 0.8x0.7 mm, TR 600ms, TE 8.6ms, two averages. Acquired with magnetization transfer weighting both on and off. Total acquisition time 8 minutes.

Supplementary Methods 3: MRI Analysis

SIENA: Two-timepoint percentage change in brain parenchymal volume was estimated with SIENA (2, 3), part of FSL (4). The 3-dimensional structural MPRAG T1 brain images entered into the SIENA analysis pipeline were first bias field corrected and lesions filled using the method described by Gelineau-Morel *et al.* (5). SIENA is a semi-automated method that begins by extracting brain and skull images from the two-timepoint whole-head MRI data (3). The two brain images are then aligned to each other (6, 7) and are resampled into the space halfway between the two. Next, tissue-type segmentation is carried out (8) in order to find brain/non-brain edge points, and then perpendicular edge displacement (between the two timepoints) is estimated at these edge points. Finally, the mean edge displacement is converted into an (global) estimate of percentage brain volume change between the two timepoints.

Voxel-Wise Edge Displacement Calculations: The process involves taking the edge displacement image between both timepoints for each subject and registering them to a standard space image before running voxelwise statistics to calculate the average change with the group using the permutation testing tool *Randomise* (part of FSL) and correcting for multiple comparisons across space.

Voxel-Based Morphology: First, structural images were brain-extracted using BET (3). Next, tissue-type segmentation was carried out using FAST4 (8). The resulting grey-matter partial volume images were then aligned to MNI152 standard space using the affine registration tool FLIRT (6, 7) followed by nonlinear registration using FNIRT. The resulting images were averaged to create a study-specific template, to which the native grey matter images were then non-linearly re-registered. The registered partial volume images were then modulated (to correct for local expansion or contraction) by dividing by the Jacobian of the warp field. The modulated segmented images were then smoothed with an isotropic Gaussian kernel with a sigma of 2 mm. Finally, a voxelwise general linear model was applied using permutation-based non-parametric testing with age and lesion masks included as nuisance regressors, correcting for multiple comparisons across space.

TBSS: First, FA images were created by fitting a tensor model to the raw diffusion data and then brain-extracted using BET (9). All subjects' FA data were then aligned to a standard space template using the nonlinear registration tool FNIRT. Next, the mean FA image was created and thinned to form a mean FA skeleton that represents the centres of all tracts common to the group. Each subject's aligned FA data was then projected onto this skeleton and the resulting data fed into voxelwise cross-subject statistics.

Reference List

1. Deoni SC, Rutt BK, Arun T, Pierpaoli C, Jones DK. Gleaning multicomponent T1 and T2 information from steady-state imaging data. Magn Reson Med. 2008 Dec;60(6):1372-87.

2. Smith SM, De Stefano N, Jenkinson M, Matthews PM. Normalized accurate measurement of longitudinal brain change. J Comput Assist Tomogr. 2001 May-Jun;25(3):466-75.

3. Smith SM, Zhang Y, Jenkinson M, Chen J, Matthews PM, Federico A, et al. Accurate, robust, and automated longitudinal and cross-sectional brain change analysis. Neuroimage. 2002 Sep;17(1):479-89.

4. Smith SM, Jenkinson M, Woolrich MW, Beckmann CF, Behrens TE, Johansen-Berg H, et al. Advances in functional and structural MR image analysis and implementation as FSL. Neuroimage. 2004;23 Suppl 1:S208-19.

5. Gelineau-Morel R, Tomassini V, Jenkinson M, Johansen-Berg H, Matthews PM, Palace J. The effect of hypointense white matter lesions on automated gray matter segmentation in multiple sclerosis. Hum Brain Mapp. 2011 Oct 5.

6. Jenkinson M, Bannister P, Brady M, Smith S. Improved optimization for the robust and accurate linear registration and motion correction of brain images. Neuroimage. 2002 Oct;17(2):825-41.

7. Jenkinson M, Smith S. A global optimisation method for robust affine registration of brain images. Med Image Anal. 2001 Jun;5(2):143-56.

8. Zhang Y, Brady M, Smith S. Segmentation of brain MR images through a hidden Markov random field model and the expectation-maximization algorithm. IEEE Trans Med Imaging. 2001 Jan;20(1):45-57.

9. Smith SM. Fast robust automated brain extraction. Hum Brain Mapp. 2002 Nov;17(3):143-55.
